# Effectiveness of Artificial Intelligence Models for Cardiovascular Disease Prediction: Network Meta-Analysis

**DOI:** 10.1155/2022/5849995

**Published:** 2022-02-24

**Authors:** Yahia Baashar, Gamal Alkawsi, Hitham Alhussian, Luiz Fernando Capretz, Ayed Alwadain, Ammar Ahmed Alkahtani, Malek Almomani

**Affiliations:** ^1^College of Graduate Studies, Universiti Tenaga Nasional (UNITEN), Selangor, Malaysia; ^2^Faculty of Computer Science and Information Systems, Thamar University, Thamar, Yemen; ^3^Department of Computer and Information Sciences, Universiti Teknologi PETRONAS, Seri Iskandar, Malaysia; ^4^Department of Electrical and Computer Engineering, Western University, London, ON, Canada; ^5^Computer Science Department, Community College, King Saud University, Riyadh, Saudi Arabia; ^6^Institute of Sustainable Energy, Universiti Tenaga Nasional (UNITEN), Selangor, Malaysia; ^7^Department of Software Engineering, The World Islamic Sciences and Education University, Amman, Jordan

## Abstract

Heart failure is the most common cause of death in both males and females around the world. Cardiovascular diseases (CVDs), in particular, are the main cause of death worldwide, accounting for 30% of all fatalities in the United States and 45% in Europe. Artificial intelligence (AI) approaches such as machine learning (ML) and deep learning (DL) models are playing an important role in the advancement of heart failure therapy. The main objective of this study was to perform a network meta-analysis of patients with heart failure, stroke, hypertension, and diabetes by comparing the ML and DL models. A comprehensive search of five electronic databases was performed using ScienceDirect, EMBASE, PubMed, Web of Science, and IEEE Xplore. The search strategy was performed according to the Preferred Reporting Items for Systematic Reviews and Meta-Analysis (PRISMA) statement. The methodological quality of studies was assessed by following the Quality Assessment of Diagnostic Accuracy Studies 2 (QUADAS-2) guidelines. The random-effects network meta-analysis forest plot with categorical data was used, as were subgroups testing for all four types of treatments and calculating odds ratio (OR) with a 95% confidence interval (CI). Pooled network forest, funnel plots, and the league table, which show the best algorithms for each outcome, were analyzed. Seventeen studies, with a total of 285,213 patients with CVDs, were included in the network meta-analysis. The statistical evidence indicated that the DL algorithms performed well in the prediction of heart failure with AUC of 0.843 and CI [0.840–0.845], while in the ML algorithm, the gradient boosting machine (GBM) achieved an average accuracy of 91.10% in predicting heart failure. An artificial neural network (ANN) performed well in the prediction of diabetes with an OR and CI of 0.0905 [0.0489; 0.1673]. Support vector machine (SVM) performed better for the prediction of stroke with OR and CI of 25.0801 [11.4824; 54.7803]. Random forest (RF) results performed well in the prediction of hypertension with OR and CI of 10.8527 [4.7434; 24.8305]. The findings of this work suggest that the DL models can effectively advance the prediction of and knowledge about heart failure, but there is a lack of literature regarding DL methods in the field of CVDs. As a result, more DL models should be applied in this field. To confirm our findings, more meta-analysis (e.g., Bayesian network) and thorough research with a larger number of patients are encouraged.

## 1. Introduction

Heart failure and related diseases are the most common cause of death in both males and females in practically all countries around the world [1]. Cardiovascular diseases (CVDs), in particular, are the main cause of death worldwide, accounting for 30% of all fatalities in the United States [2] and 45% in Europe, while costing the European Union €210 billion each year [3]. Despite substantial advances in diagnostic procedures over the last 50 years, cardiologists, primary care physicians, and other healthcare providers face tremendous challenges in the early detection and diagnosis of heart disease [1]. Physicians are also trained to diagnose CVD based on a patient's medical history, basic ratings, physical tests, and biomarkers, which are interpreted according to their own clinical experience. They then match each patient to the conventional taxonomy of medical diseases based on their subjective comprehension of the medical literature. This practice is becoming more error-prone and inefficient [4]. In addition, as the proficiency of cardiovascular techniques in collecting large volumes of data continues to improve, physician's jobs become more complex. As a result, medical treatments must be simple to use, fast, and automated, as well as highly accurate to improve patient well-being while also decreasing healthcare costs and reducing deaths from CVD.

Artificial intelligence (AI) approaches such as machine learning (ML) and deep learning (DL) models are playing an important role in the advancement of heart failure (HF) therapy. However, clinical HF care is currently challenged with real-world problems, including the need for cost savings in prevention and treatment, high readmission and mortality rates, insufficient patient care, and overutilization [5, 6]. Applying AI-based predictive modeling can address these problems; however, constructive collaborations between data scientists and medical professionals are essential to supporting the clinical effectiveness of automation and diagnostic systems through AI [7].

Due to enormous advancements in data processing and warehousing capabilities, ML is becoming very popular and is considered a reliable method for combining clinical data and physician's reports from electronic medical records (EMRs) to improve the accuracy of a wide range of medical tasks [8]. DL on the other hand has emerged as a robust solution for medical tasks, including image classification, image segmentation, and natural language processing (NLP) as a result of big data and improved computational capability of graphical processing units (GPUs). Among the most common AI models used in CVD are logistic regression (LogR) [9,10], support vector machine (SVM) [11], gradient boosting machine (GBM) [12–14], random forest (RF) [13, 15], artificial neural network (ANN) [15, 16], and convolutional neural network (CNN) [17, 18].

Several studies have examined the AI models in predicting different outcomes for CVDs. Damen et al. [19] performed a systematic review that described the construction or external validation of a multivariable model for predicting CVD risk in the general population and concluded that there are too many models for predicting CVD [19]. The effectiveness of most of the models is doubtful because of shortcomings in methodology and a lack of external validation studies. The work also concluded that rather than introducing new CVD risk prediction models, future research should concentrate on validating and comparing current models to see how they can be improved. Al'Aref et al. [20] reviewed the current ML approaches for building inferential and predictive data-driven models within CVD. The study identifies various areas where ML can be used, including echocardiography, electrocardiography, and newly discovered noninvasive imaging modalities such as coronary artery calcium scoring and coronary computed tomography angiography. The study also identified the limitations of the current ML algorithms in the field of CVD, underlining the necessity for AI to integrate temporal and spatial data into composite patient-centric information that improves the value of medical treatments.

A recent meta-analysis was performed by Krittanawong et al. [21] to assess the ability of different ML models to predict stroke, heart failure, cardiac arrhythmias, and coronary artery disease. This work found that the predictive capabilities of boosting algorithms and SVM are promising in the field of CVD.

Assessing the current literature related to DL methods, we found that only a few reported the summary values of the treatments and the statistics of the patient's family history, which makes it difficult for readers to understand the best algorithm. To our knowledge, none of the studies have performed a meta-analysis on the effectiveness of both ML and DL for the prediction of heart failure, with the exception of one study [21], which focused on ML only. Hence, filling this research gap allows an important contribution to be made. The aim of this study was to perform a network meta-analysis on both ML and DL models on 285,213 patients with CVD in predicting four outcomes (heart failure, stroke, diabetes, and hypertension), which none of the literature has done before.

A comprehensive understanding of the factors related to treatment outcomes in patients with heart failure, stroke, hypertension, and diabetes is required to create effective strategies to enhance these treatments. However, the current studies of patients with the aforementioned diseases have a number of drawbacks that restrict the expediency of their findings. One of these drawbacks is the failure to combine DL and ML, which we have addressed by carrying out this network meta-analysis. The findings of this study have a number of implications for assisting policymakers and medical professionals on how to understand the data and apply different AI models to predict outcomes in patients with CVD.

The remainder of the study is structured as follows: Section 2 describes our method for conducting the network meta-analysis, which includes the data extraction, risk-of-bias assessment, and the statistical analysis.  Section 3 presents the findings of the study, and section 4 discusses and evaluates the findings. Lastly, section 5 presents the conclusions of the study.

## 2. Materials and Methods

To achieve the aims of this study, a network meta-analysis was carried out. This was accomplished by following the guidelines of PRISMA [22], which include the eligibility criteria, search strategy, selection of studies, data extraction, risk-of-bias assessment, and data analysis. In addition, this work used the quality assessment of QUADAS-2 to evaluate the quality of the studies that were included [23].

### 2.1. Eligibility Criteria

The target population was patients (adults >18, male/female), suffering from CVD. The qualified interventions were both DL and ML models that predict CVD. The outcomes of the network meta-analysis are identifying patients affected with heart failure, diabetes, hypertension, and stroke.

To obtain the most recent published works in the field of AI and CVD, only studies published from 2016 to April 2021 were included. There were no limitations on the country in which studies were conducted. Regarding the study design, observational cohort and experimental studies were included. The current work also included articles from conference proceedings, peer-reviewed journals, and repositories of electronic prints. We excluded any sort of reviews (systematic or traditional), proposals, dissertations, editorials, conference abstracts, and studies that were in languages other than English.

### 2.2. Search Strategy

A comprehensive and systematic search of five electronic databases was performed. These include ScienceDirect, EMBASE, PubMed, Web of Science, and IEEE Xplore. We also searched Google Scholar to identify relevant studies. However, the results were sorted for Google Scholar based on relevancy and date (2016–2021). Then, only the first 300 results (30 pages) were screened. The search was conducted in March 2021. Furthermore, a backward and forward reference searching was carried out, where both reference lists and the work cited from the selected studies were screened. It is important to remember that each electronic source has its own particular features, which meant that the search strategy had to be adapted and modified accordingly. For example, the use of Google Scholar's function “cited by” was very useful in identifying relevant articles.

The search terms used were related to AI interventions (e.g., ML and DL) and the targeted population (e.g., adult patients with CVD). Our search keywords were adjusted and tested in various online databases as follows: [“cardiovascular” OR “heart disease” OR “heart failure”] AND [“prediction” OR “detection” OR “identification”] AND [“artificial intelligence”] OR [“deep learning” OR “machine learning”].  Table 1 summarizes all the keywords and terms used in the search.

### 2.3. Study Selection

**Figure 1:**
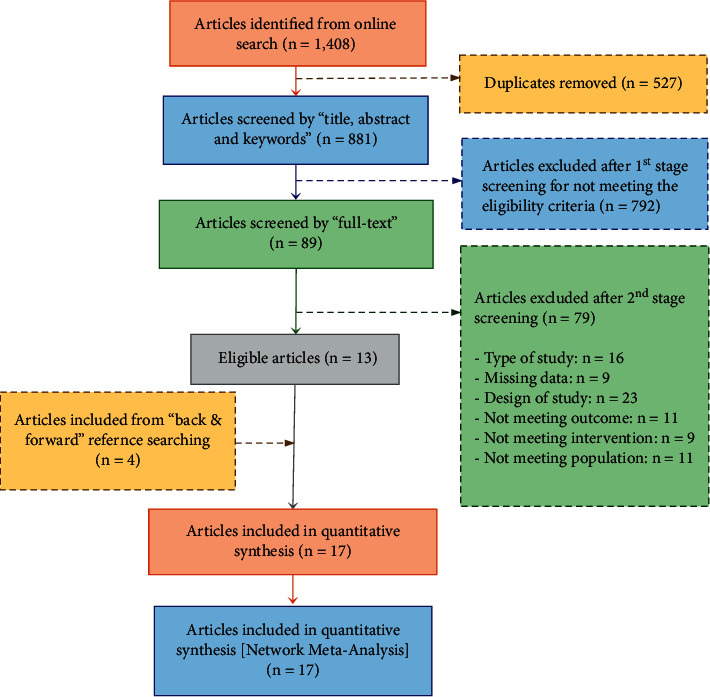
Study screening and selection flowchart.

Following the search strategy, two stages were undertaken in the selection process. First, the titles, abstracts, and keywords of all records were screened (see Figure 1). Any records that did not fulfill the inclusion criteria were excluded. If there were any doubts, the studies were considered for the second stage, which is the full-text screening. Two authors (Y. B. and G. A.) of this study have independently performed both stages of the selection process. Any differences between the authors were sorted out through consensus.

### 2.4. Data Extraction

EndNote X20 software was used to extract basic publication records such as title, authors, date, DOI, and publisher. One author (H.A.) compiled the data elements from the studies that were included in Microsoft Excel 2019, where two other authors (L.F.C. and A.A.) independently validated them. The data items extracted from each of the selected studies include author(s), publication year, number of participants, type of AI used (e.g., DL and ML), outcomes, analytical model or algorithm, indication, comparisons, and data sources.

### 2.5. Risk-of-Bias Assessment

The QUADAS-2 tool [23] was used to assess the quality of the studies. Two authors (A.A.A. and M.A.) independently assessed the following four domains for the risk of bias: (1) patient selection; (2) index test; (3) reference standard; and (4) flow and timing. The differences were settled through group discussions until reaching a consensus.

**Figure 2:**
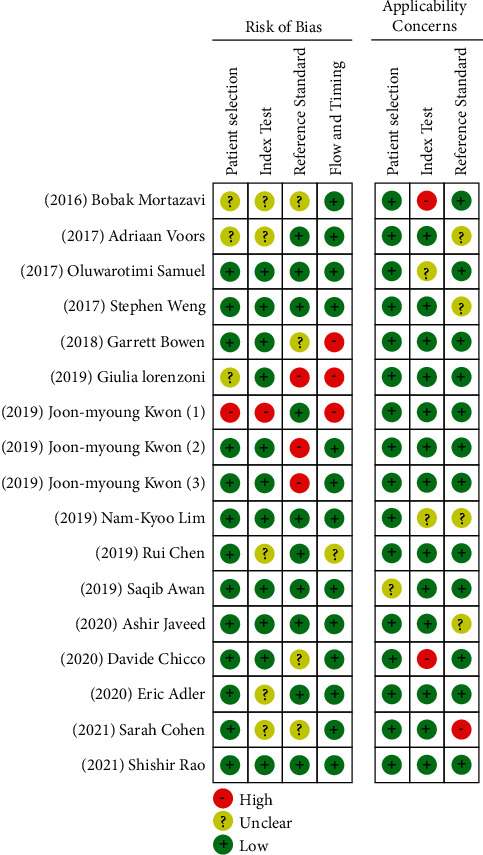
Graph showing risk of bias for each study.

**Figure 3:**
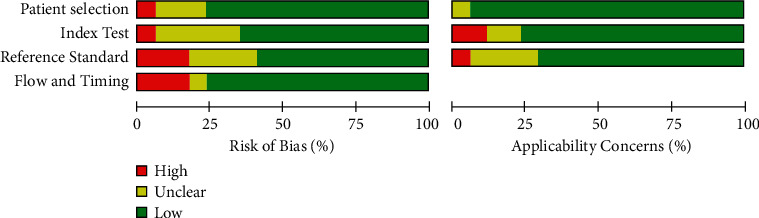
Graph showing risk of bias across all studies.

The studies chosen were graded on each domain as being of “high,” “low,” or “unclear” risk. In addition, RevMan 5.4 was used to produce the results, which are shown in Figures 2 and 3, along with the author's judgments regarding “risk of bias” in each, and across all studies. The use of QUADAS-2 tool, domains, and ratings is further described in the supplementary material (available here).

### 2.6. Data Analysis

Categorical data were reported as number (mean, standard deviation, and percentage). The number of patients, method of AI used in the prediction, and the score of the control group and the treatment group for each study were compiled in an Excel sheet. We conducted a network meta-analysis on studies of a good quality that reported both DL and ML algorithms for predicting CVD (e.g., heart failure, stroke, diabetes, and hypertension). To produce good and reliable results, a proportion network meta-analysis was performed for the individual outcome using *R* statistical software version 4.0.2, which generated the forest plot that shows the proportion (P), confidence interval (CI), and the heterogeneity measured with (*I*^2^). The random-effects model was selected because the true effect size underlying all studies was stochastic. The individual forest plots were reported as P and CI, the pooled forest plot was reported as internal rate of return (IRR), and lastly the league table was reported as odds ratio (OR).

The sensitivity and specificity analysis, which measured the percentage of the identified participants with the four outcomes and the network plot, was also analyzed using *R* statistical software version 4.0.2. The quality of the studies was assessed using RevMan version 5.4.

## 3. Results and Analysis

### 3.1. Search Results of the Included Studies

We identified 1,408 articles from our initial online database search (see Figure 1). The articles removed after identifying duplicates (*n* = 527) resulted in a total of 881 unique articles that were screened based on the first stage (title, abstract, and keyword). 792 articles were excluded for not meeting the eligibility criteria. The remaining articles (*n* = 89) were screened based on the second stage (full text). Of these, 13 articles were included and met all the eligibility criteria. Following that, a total of four additional articles were found and included from backward (*n* = 3) and forward (*n* = 1) reference searching. Overall, 17 articles were included in both qualitative synthesis and the network meta-analysis.

### 3.2. Descriptions of the Included Studies

17 selected studies were assessed thoroughly to extract the following data: authors and year, type of interventions and algorithms, indication and outcomes, sample size, data source, comparison, and number of patients with heart failure, stroke, hypertension, and diabetes mellitus. The results of data extraction and characteristics of each individual study are provided in the supplementary material (available here).

The design of the selected studies was observational (14/17, 82%) as shown in Table 2, while the remaining studies were experimental (3/17). Among the 14 observational studies, half were prospective cohorts and the other half were retrospective cohorts. The year of publication ranged from 2016 to 2021, with 41 percent of studies published in 2019 (7/17). The research was conducted in eight countries, including the United States, United Kingdom, Canada, China, Italy, Netherlands, Australia, and Korea. Six of 17 (6/17) studies were conducted in both China and the United States equally, while 4 of 17 were performed in Korea.

Regarding the population and sample size, only one study reported less than 100 participants (e.g., patients), while 3 of 17 studies ranged from 101 to 1,000 participants, and 5 of 17 were between 1001 and 10,000. The majority of studies (*n* = 7, 41%) reported a sample size between 10,001 and 60,000. Only one study had a size above 60,000 participants (see Table 2). The overall sizes ranged from 98 to 100,071. Patient's sex (female) was reported in more than 40% of the studies (*n* = 10). Furthermore, only one study reported a rate of less than 10% female participants. The overall percentage of females in the studies ranged from 2 to 54.

The selected studies examined one of the two interventions, machine learning (13/17, 76.5%) or deep learning (4/17, 23.5%). Both were used to predict CVD and HF risk (5/17), HF hospitalization (2/17), HF readmission (2/17), and HF mortality (6/17), while two other studies have predicted several outcomes (e.g., HF mortality with both readmission and hospitalization). Further details on each selected study are available in the supplementary material (available here).

### 3.3. Risk of Bias in the Included Studies

We assessed the quality of studies by following the guidelines of QUADAS-2 (see Figures 2 and 3 and the supplementary material (available here)). Among all of the 17 selected studies [9–16, 24–32], only 3 studies [16, 24, 30] showed “high risk” of bias on the “applicability concerns,” and the majority of studies were “low risk.” Regarding the domain of “patient selection,” which addresses the question “Could the selection of patients or study participants have introduced bias?” among the studies that used ML algorithms in predicting heart failure, only three studies [9, 11, 30] reported “unclear risk” of bias, while others reported a low risk. For the DL algorithms, only one study [28] compared the DL with a traditional logistic regression and reported a “high risk,” while others [26, 27, 31] listed a “low risk.”

In terms of the “index text” domain, which addresses the question “Could the conduct or interpretation of the index test have introduced bias?”, none of the studies that used ML answered a “high risk” of bias, while only five studies [9, 12, 13, 24, 30] reported “unclear risk.” As for DL studies, one has indicated “high risk” [28], while others reported “low risk” (see Figure 1).

Regarding the “reference standard” domain, four ML studies [10, 16, 24, 30] reported “unclear risk” of bias, and one study [11] answered “high risk,” while the others reported “low risk.” For DL algorithms, two studies [26, 27] answered “high risk” of bias, and the remaining studies reported “low risk.” Figure 2 shows the risk of bias across all of the included studies for both DL and ML methods.

### 3.4. Results of the Network Meta-Analysis

A network meta-analysis using a random-effects model was performed using the dataset in the supplementary material (available here) for each of the four outcomes (e.g., diabetes, stroke, heart failure, and hypertension). The coding of the final analysis is also provided in the supplementary material (available here). The analysis was only performed on the number of studies (>5) and for the outcome that reported the study (<5). The overall results of the network meta-analysis are summarized in Table 3, which is a league table showing the results by comparing all AI models.

#### 3.4.1. Prediction of Heart Failure

**Figure 4:**
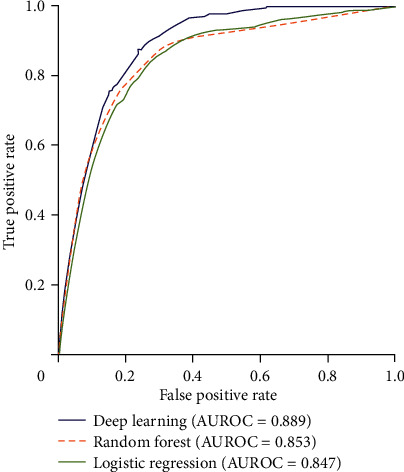
Network curve showing the results of DL and ML algorithms.

For heart failure prediction, two DL observational studies [26, 28] reported a total of 108,584 patients. One study [27] used and compared the DL model with LogR and RF. After the comparison, we found that the area under the receiver operating characteristic curves (AUROCs) for the identification of best algorithm in heart failure was 0.843 (95% CI, 0.840–0.845) as illustrated in Figure 4. This outperformed those of LogR (0.800 [0.797–0.803], 0.847 [0.844–0.850]) and RF (0.807 [0.804–0.810], 0.853 [0.850–0.855]).

Furthermore, 10 ML studies reported a total of 94,714 patients with heart failure. Of these, two prospective cohort studies [16, 30] and one experimental study [11] used SVM for the prediction of heart failure, and two experimental studies [25, 32] used ANN; two retrospective cohort studies [9, 10] used LogR, and three studies [12–14] used GBM. The prediction of heart failure was associated with the result, which shows that GBM models achieved an average prediction accuracy of 91.10%, which is 4.40% higher than other models (e.g., ANN, SVM, and LogR).

**Figure 5:**
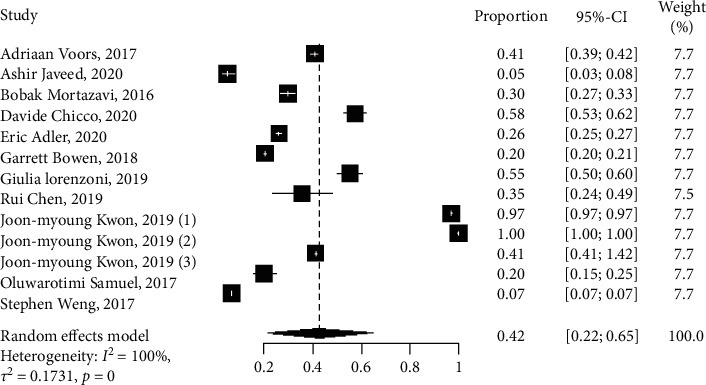
Network meta-analysis forest plot of studies reporting the total number of patients with heart failure.

All studies that reported heart failure outcomes were pooled together, and a random-effects forest plot (Figure 5) shows that the results were statistically significant (*I*^2^ = 100%, *p* < 0.05). The proportion of the number of samples and the weight of each study are shown in the plot (Figure 5), and only one DL observational study [28] has a proportion equal to 1. However, with a sensitivity value of 97.4% and a specificity value of 19%, we were able to identify a higher percentage of the patients with heart failure.

#### 3.4.2. Prediction of Diabetes

**Figure 6:**
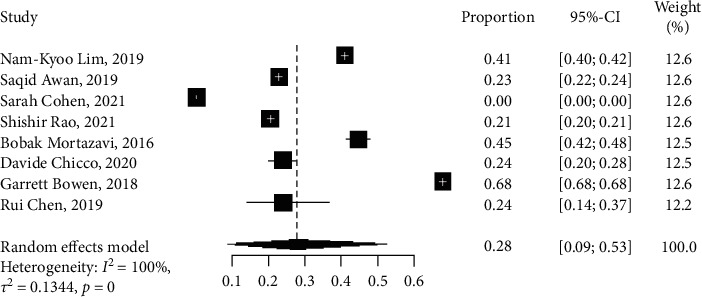
Network meta-analysis forest plot of studies reporting the number of patients with diabetes.

Diabetes outcomes were reported in seven ML studies [10, 13, 15, 16, 24, 29, 30] and one DL study [31], with a total of 75,265 patients. Among these, two studies [13, 30] applied GBM models and another two [15, 16] used ANN methods. The results in Table 3 show that ANN outperformed GBM and other models in the prediction of diabetes with an odds ratio (OR) and CI of 0.0905 [0.0489; 0.1673].  Figure 6 shows that the results were statistically significant (*I*^2^ = 100%, *p* < 0.05). The proportion of the number of samples and the weight of each study are shown in the plot (Figure 6). A DL study [31] has a proportion value of 0. However, with a sensitivity value of 88.5% and a specificity value of 14%, this implies that we were able to identify a higher percentage of patients with diabetes.

#### 3.4.3. Prediction of Hypertension

**Figure 7:**
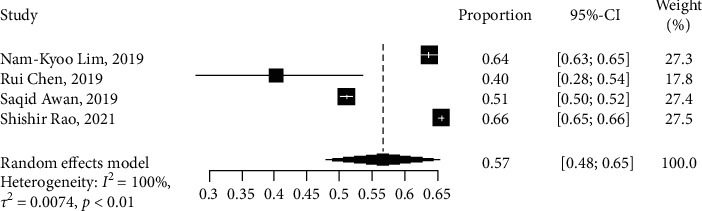
Network meta-analysis forest plot of study that reported number of patients with hypertension.

The hypertension outcome was reported in four observational studies with a total of 76,100 patients. Three studies were related to ML method [13, 15, 29], and one study applied the DL method [31]. Of these, two studies [13, 15] used and compared RF with LogR, GBM, and LASSO regression. We were unable to perform analysis due to the limited number of studies in the observational groups (<5); however, RF results performed well in the prediction of hypertension with OR and CI of 10.8527 [4.7434; 24.8305]. The sensitivity value of 69.7% and the specificity value of 17% show that we were able to identify a higher percentage of people with hypertension. All four studies that reported the outcome of hypertension were pooled together, and a random-effects forest plot in Figure 7 shows that the results were statistically significant (*I*^2^ = 100%, *p* < 0.05). The proportion of the number of samples and the weight of each study are shown in the plot (Figure 7).

#### 3.4.4. Prediction of Stroke

**Figure 8:**
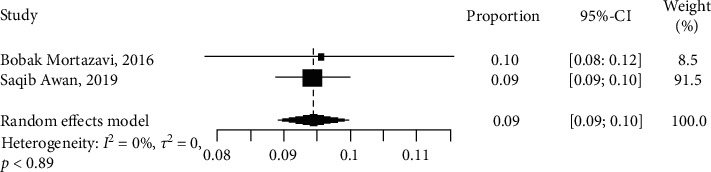
Network meta-analysis forest plot of studies reporting the number of patients with stroke.

Only two ML studies reported the stroke outcome with a total number of 10,821 patients. Of these, one study [30] used SVM, GBM, and RF algorithms, while the other study [15] used traditional ML models such as LogR. We could not perform analysis because we had too few studies (<5) for the model. However, Table 3 shows that SVM performed better for the prediction of stroke with OR and CI of 25.0801 [11.4824; 54.7803].  Figure 8 shows that the differences in the results, methodology, and the number of patients used in the study were not statistically significant (*I*^2^ = 0%, *p* > 0.05). The proportion of the number of samples and the weight of each study are shown in the plot (Figure 8). Nonetheless, a sensitivity value of 74.1% and a specificity value of 21% imply that we were able to identify a higher percentage of patients with stroke.

**Figure 9:**
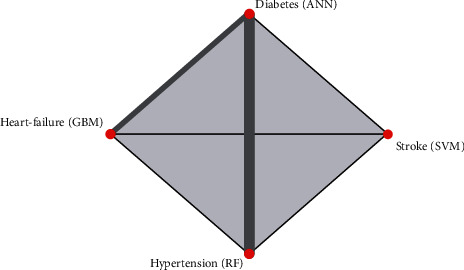
Network plot of the four outcomes.

In Figure 9, the network plot affirmed the above results, indicating the best algorithm for each outcome. It can be seen that GBM performs well for the prediction of heart failure, ANN for the prediction of diabetes, RF for the prediction of hypertension, and SVM for the prediction of stroke. The summary of the complete results can be found in Table 3.

**Figure 10:**
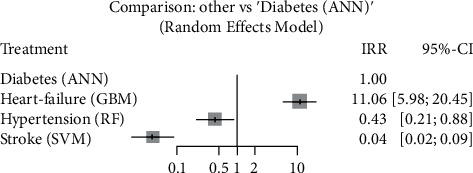
Pooled network forest plot for the four outcomes.

In Figure 10, the pooled network meta-analysis of all studies shows that the overall effect was statistically significant (*p* < 0.05), and the heterogeneity between the subgroups was also significant (*p* < 0.05) with a quantifying heterogeneity effect of 91.86%.

### 3.5. Publication Bias

**Figure 11:**
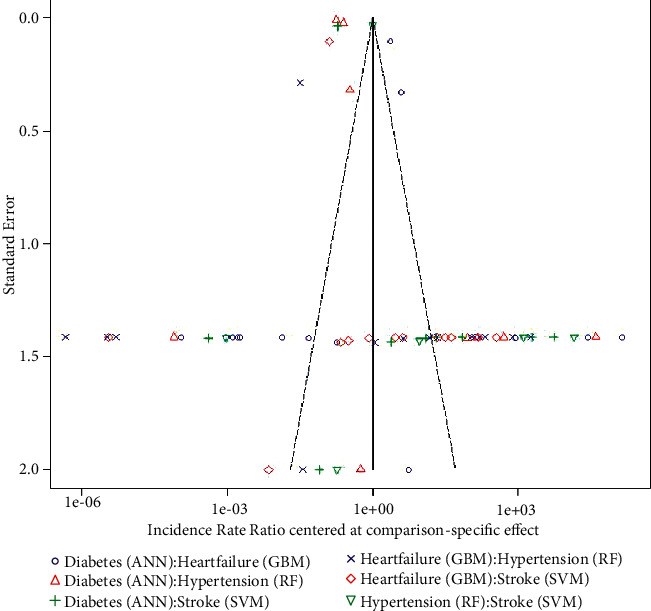
Network meta-analysis funnel plot indicating the DL and ML models for the four outcomes.

Figure 11 presents the network meta-analysis funnel plot, which shows the results of deep learning and machine learning in predicting the four indications (heart failure, stroke, diabetes, and hypertension). The plot is a graph of standard error against the incidence rate ratio, the different colored symbols and shapes indicate how the studies are spread out symmetrically, and the symmetric nature of the plot shows no indication of publication bias across all studies.

## 4. Discussion and Assessments

### 4.1. Key Findings

It is very important to understand the background, causes, and factors associated with heart failure to develop effective interventions that can enhance medication adherence [33, 34]. However, the majority of medication adherence studies in patients with the diseases mentioned above have had several drawbacks that degrade the usefulness of their results. One of these drawbacks is the failure to combine both DL and ML models to examine medication adherence. This study addressed this problem by conducting a network meta-analysis to determine medication adherence predictors for patients with heart failure, stroke, hypertension, and diabetes. In most of the studies that used ML, the overall analysis showed an AUC of 0.8–0.9 s for the prediction of CVD. Also, looking at the subgroup analysis according to the literature, ML models seem to perform well, with AUC values between 0.80 and 0.90 for the prediction of heart failure and stroke.

To date, none of the current literature has done a network meta-analysis for both ML and DL algorithms. However, we found one study by Liu et al. [35], which used DL models with a methodology similar to our study. The authors of [35] compared and evaluated the diagnostic performance of several DL algorithms based on medical imaging (two studies were related to cardiology). The work concluded that the DL models were effective and promising, yet various methodological barriers related to accuracy at the clinician level were identified. Although our analysis revealed that GBM is very effective in the prediction of heart failure, further work comparing machine learning and human expertise is needed.

According to our network analysis, we found that DL interventions produced better performance than ML for predicting heart failure in terms of their AUC, which is reported in Figure 4. Through the comparison of AUC, GBM algorithms seem to perform well. According to Mayr et al. [36] and Bühlmann et al. [37], GBM has been increasingly utilized in modern biomedicine. However, to implement it in a clinical practice, the essential stages of designing a model and interpretation need to be uniform [38].

Regarding the prediction of stroke, our analysis revealed that SVM and RF yielded a good value for AUC. Both SVM and RF showed promising results for addressing the clinical matters, but SVM had a better performance in the prediction of stroke with IRR and a standard deviation of 25.0801 [11.4824; 54.7803] in patients with stroke. This might be because of the linear discrete data that fit better within enhanced generalization. Noble [39] stated that SVM is more effective in realizing unknown patterns in complex clinical datasets, when compared to other ML models.

For diabetes prediction, we were unable to perform a network meta-analysis due to the limited number of studies in the observational cohort for both DL and ML models. However, based on the analysis we performed and the results from Table 3, ANN outperformed all other predictive models in identifying patients with diabetes, particularly in a study reported by [16], and also as confirmed by the network plot in Figure 9. ANN is one of the most powerful algorithms for the prediction of CVD, and our study found that it can also be helpful for the prediction of diabetes. In addition, it can be implemented in the electronic medical records (EMRs) to assist its application in the clinical system and minimize mortality rates.

Regarding the prediction of hypertension, we were also unable to carry out a network meta-analysis due to the problems reported above. However, as illustrated in Figure 10, the FR models performed well in the prediction of hypertension, which was statistically significant with IRR of 0.43 (0.21, 0.88) and odds ratio (OR) of 10.8527 [4.7434; 24.8305], as can be seen in Table 3.

Lastly, Figure 11 presented the funnel plot of the network meta-analysis showing the permutation of the algorithm along with the variables. A symmetric funnel plot shows no evidence of publication bias across any of the studies selected.

### 4.2. Limitations and Strengths

Our study has a few limitations. The first limitation of this work was the small number of studies for some outcomes, which made it difficult to perform network meta-analysis; the number of studies required for a network analysis has been set at >5. Secondly, we do not have access to some of the articles needed for the final analysis due to location and access restrictions. Thirdly, some of the studies did not report the overall mean and standard deviation for the control and experimental groups, which made it difficult to use Cohen's *D* to compute the effect size and standard needed for the pooled network analysis. Finally, our data were mainly based on the DL and ML methods, and according to Berkson's bias [40], if other interventions apart from DL and ML are not included, it may lead to excessive or inaccurate approval. Both ML and DL have been extensively applied in several areas, including recognition, medicines, bioinformatics, and reliability evaluation for survival analysis of various chronic illnesses [41]. Many studies have approved the use of SVM for the prediction of heart failure, but this study investigated the use of GBM as an accurate predictor of heart failure.

One of the main strengths and contributions of our study is being among the first to perform a network meta-analysis to assess both DL and ML methods in the prediction of CVD, on a total of 285,213 patients with four outcomes, namely heart failure, stroke, diabetes, and hypertension.

## 5. Conclusion

The effectiveness of artificial intelligence models (DL and ML) in the prediction of cardiovascular diseases were assessed in this study. The network meta-analysis included 17 studies with a total of 285,213 patients from 2016 to 2021. Our findings suggested that there are numerous limitations to overcome before DL and ML models can be fully implemented in medical practice. DL models showed more promising results than ML. GBM, on the other hand, is gaining more popularity and is already widely used in CVD prediction. However, our study focused on four outcomes, heart failure, stroke, diabetes, and hypertension, as well as selecting the appropriate algorithm for each outcome.

Even with the difficulties of validating observational studies, the human expert's comparison, and the reporting of evaluation matrices within the correct medical context, our study found that GBM performed well in the prediction of heart failure, SVM showed good results in the prediction of stroke, ANN yielded good results in diabetes prediction, and RF performed well in the prediction of hypertension.

Other scholars who wish to carry out similar work are advised to perform a Bayesian network meta-analysis approved with a suitable prior, likelihood, and posterior distribution, as well as focusing more on DL models for the same or different outcomes related to cardiovascular diseases.

## Figures and Tables

**Table 1:** Keywords and terms used in the search.

No.	Category/terms	Keywords
1	Cardiovascular diseases	Heart failure, heart failure risk, acute heart failure, congenital heart disease, heart disease, heart attack, chronic heart disease, coronary heart disease
2	Prediction	Detection, identification, predict, detect, identify
3	Artificial intelligence	Deep learning, machine learning, AI, DL, ML, ANN, CNN

**Table 2:** Study characteristics (*n* = 17).

Characteristics	*n* (%)
*Study design*	
Observational	14 (82.4)
Experimental	3 (17.6)
*Year of publication*	
2016	1 (5.9)
2017	3 (17.6)
2018	1 (5.9)
2019	7 (41.2)
2020	3 (17.6)
2021	2 (11.8)
*Country*	
United States	3 (17.6)
Canada	2 (11.8)
United Kingdom	2 (11.8)
Italy	1 (5.9)
Netherlands	1 (5.9)
Australia	1 (5.9)
China	3 (17.6)
Korea	4 (23.5)
*Population*	
Sample size	
<100	1 (5.9)
101–1000	3 (17.6)
1001–10,000	5 (29.4)
10,001–60,000	7 (41.2)
>60,000	1 (5.9)
*Gender (female)* (%)	
<10	11 (5.9)
11–29	3 (17.6)
30–39	3 (17.6)
>40	10 (58.8)
*Intervention*	
Machine learning	13 (76.5)
Deep learning	4 (23.5)
*Predicted outcomes*	
CVD and HF risk	5 (29.4)
HF hospitalization	2 (11.8)
HF readmission	2 (11.8)
Mortality	6 (35.3)
Both mortality and HF readmission	1 (5.9)
Both HF mortality and HF hospitalization	1 (5.9)

**Table 3:** League table showing the result of the network meta-analysis.

**Diabetes (ANN)**	0.1796 [0.0934–0.3453]	1.7786 [0.8496; 3.7234]	12.7319 [5.5161; 29.3872]
0.0905 [0.0489; 0.1673]	**Heart failure (GBM)**	27.7774 [12.9133; 59.7514]	141.5198 [64.9910; 308.1636]
2.3109 [1.1331; 4.7130]	25.5489 [12.7595; 51.1574]	**Hypertension (RF)**	5.4764 [2.2916; 13.0876]
25.0801 [11.4824; 54.7803]<	278.2743 [133.6422; 575.2751]	10.8523 [4.7434; 24.8305]	**Stroke (SVM)**

Odds ratios (OR) and 95% CI. OR ＞1 means the top-left algorithm is better.

## Data Availability

The data supporting this network meta-analysis are from previously reported studies, which have been cited. The processed data used to support the findings of this study are included within the supplementary information files.

## Supplementary Materials

Supplementary Materials File 1: QUADAS-2 tool (DOCX file, 13.9 KB). File 2: characteristics of the selected studies (DOCX file, 24.0 KB). File 3: dataset used in the network meta-analysis (DOCX file, 13.6 KB). File 4: coding of the network meta-analysis using R (DOCX file, 13.6 KB).
