# Supplementary material for: Effectiveness of Artificial Intelligence Models for Cardiovascular Disease Prediction: Network Meta-Analysis
Source: Comput Intell Neurosci. 2022 Feb 24;2022:5849995. doi: 10.1155/2022/5849995 (PMC8894073; doi:10.1155/2022/5849995)
Supplement: Supplementary Materials — File 1: QUADAS-2 tool (DOCX file, 13.9 KB). File 2: characteristics of the selected studies (DOCX file, 24.0 KB). File 3: dataset used in the network meta-analysis (DOCX file, 13.6 KB). File 4: coding of the network meta-analysis using R (DOCX file, 13.6 KB). [file 5849995.f1.zip › 5849995.f1/File 1. The QUADAS-2 tool..docx]

Supplementary 1. The QUADAS-2 tool.

| **Domain** | **Patient Selection** | **Index Test** | **Reference Standard** | **Flow and Timing** |
| --- | --- | --- | --- | --- |
| Description | Describe methods of patient selection.  Describe included patients (prior testing, presentation, intended use of index test and setting). | Describe the index test and how it was conducted and interpreted. | Describe the reference standard and how it was conducted and interpreted. | Describe any patients who did not receive the index test(s) and/or reference standard or who were excluded from the 2x2 table (refer to flow diagram).  Describe the time interval and any interventions between index test(s) and reference standard. |
| Signaling questions (yes/no/unclear) | - Was a consecutive or random sample of patients enrolled?  -Did the study avoid in- appropriate exclusions?    - Was a case-control design avoided? | - Were the index test results interpreted without knowledge of the results of the reference standard?  - Were the methods for CSF collection and handling appropriately described?  - Do the he studies forecasts the exclusion of blood-contaminated samples on the basis of an accurate cut –off? | - Is the reference standard likely to correctly classify the target condition?  - Were the reference standard results interpreted without knowledge of the results of the index test? | - Was there an appropriate interval between index test(s) and reference standard?  - Did all patients receive a reference standard?  - Did all patients receive the same reference standard?  - Were all patients included in the analysis? |
| Concerns regarding applicability: High/low/ unclear | Are there concerns that the included patients do not match the review question? | Are there concerns that the index test, its conduct, or interpretation differs from the review question? | Are there concerns that the target condition as defined by the reference standard does not match the review question? |  |
