# Supplementary material for: Effectiveness of Artificial Intelligence Models for Cardiovascular Disease Prediction: Network Meta-Analysis
Source: Comput Intell Neurosci. 2022 Feb 24;2022:5849995. doi: 10.1155/2022/5849995 (PMC8894073; doi:10.1155/2022/5849995)
Supplement: Supplementary Materials — File 1: QUADAS-2 tool (DOCX file, 13.9 KB). File 2: characteristics of the selected studies (DOCX file, 24.0 KB). File 3: dataset used in the network meta-analysis (DOCX file, 13.6 KB). File 4: coding of the network meta-analysis using R (DOCX file, 13.6 KB). [file 5849995.f1.zip › 5849995.f1/File 2. Characteristics of the selected studies..docx]

Supplementary 2. Characteristics of the selected studies.

| **1^st^ Author, (year)** | **Intervention** | **Algorithm** | **Sample (n)** | **Experiment Mean (SD)** | **Control Mean (SD)** | **Compared with** | **Outcome** | **Data Source** |
| --- | --- | --- | --- | --- | --- | --- | --- | --- |
| Adriaan Voors, (2017) | ML | LogR | 4,254 | 1.73 | 1.52 | - | HF | 1000 bootstrap samples. |
| Ashir Javeed, (2020) | ML | ANN | 297 | 243.49 ± 53.58 | 250.73 ± 49.83 | DNN | HF | Cleveland online heart disease database. |
| Bobak Mortazavi, (2016) | ML | GBM, SVM, RF | 977 | 1.40(0.77) and 25.2(17.8) |  | LogR | HF, Diabetes, Stroke | Tele-HF. |
| Davide Chicco, (2020) | ML | ANN | 299 | 40.27(10.86) and 1.19(0.65) | 38.08(11.83) and 1.84(1.47) | RF, DT, NB | HF, Diabetes | Available under the Creative Commons Attribution 4.0 International (CC BY 4.0). |
| Eric Adler, (2020) | ML | BDT | 5,822 | 1.2 (0.7) | 1.4 (0.9) | AdaBoost | HF | University of California, San Diego; UCSF, University of California, San Francisco |
| Garrett Bowen, (2018) | ML | LogR | 66,385 | 9.2 6 (28.3) | 7.3 6 (18.3) | - | HF, Diabetes | VA External  Peer Review Program (EPRP). |
| Giulia Lorenzoni, (2019) | ML | GBM, SVM, ANN | 380 | 0.800/1.000/1.208 | 0.810/1.070/1.450 | LogR, RF | HF | Gestione Integrata dello Scompenso Cardiaco (GISC). |
| Joon-myoung Kwon (1), (2019) | DL | - | 25,776 | 55.8 ± 13.9 | 44.2 ± 17.9 | RF, LogR | HF |  |
| Joon-myoung Kwon (2), (2019) | DL | - | 2,165 | 1.8±1.3 | 1.9±1.7 | RF, LogR, SVM, Bayesian Network | HF | The Korean Acute Heart Failure (KorAHF). |
| Joon-myoung Kwon (3), (2019) | DL | - | 22,765 | 33.75±14.31 | 27.97±11.08 | RF, LogR | HF | The Sejong General Hospital Institutional Review Board (IRB) |
| Nam-Kyoo Lim, (2019) | ML | Multiple LogR | 4,566 | 133.1(29.6) | 126.5(32.2) | - | Diabetes, Hypertension | The Korean Acute Heart Failure (KorAHF). |
| Oluwarotimi Samuel, (2017) | ML | Hybrid ANN | 303 | 0.86 ± 0.178 | 0.63 ± 0.51 | Fuzzy | HF | Online data mining repository of the University of California, Irvine (UCI). |
| Rui Chen, (2019) | ML | GBM | 98 | 94 ± 32 | 85 ± 21 | NB, RF | Diabetes, Hypertension | Patients with severe DCM (LVEF < 35%) from two centers. |
| Saqib Awan, (2019) | ML | ANN | 10,757 | 1950 (23.7) | 656 (25.8) | LogR, RF, LASSO R | HF, Diabetes, Hypertension, Stroke | The linked Hospital Morbidity Data Collection. |
| Sarah Cohen, (2021) | ML | LogR | 29,991 | - | - | - | Diabetes | The Quebec CHD database. |
| Shishir Rao, (2021) | DL |  | 100,071 | 0.13(0.10) | 0.12(0.11) |  | Diabetes, Hypertension | UK Clinical Practice Research Data link (CPRD). |
| Stephen Weng, (2017) | ML | ANN | 378,256 | 91.9 (17.3) | 87.6 (16.0) | LogR, RF, GBM | HF | The Clinical Practice Research Data link (CPRD). |

*GBM: Gradient Boosting model, SVM: Support vector machine, BDT: Boosted decision tree, ANN: Artificial neural network, RF: Random forest, DT: Decision tree, NB: Naïve bayes, LogR: Logistic regression, DNN: Deep neural network, ML: Machine learning, DL: Deep learning, HF: Heart failure*
