# Supplementary material for: Effectiveness of Artificial Intelligence Models for Cardiovascular Disease Prediction: Network Meta-Analysis
Source: Comput Intell Neurosci. 2022 Feb 24;2022:5849995. doi: 10.1155/2022/5849995 (PMC8894073; doi:10.1155/2022/5849995)
Supplement: Supplementary Materials — File 1: QUADAS-2 tool (DOCX file, 13.9 KB). File 2: characteristics of the selected studies (DOCX file, 24.0 KB). File 3: dataset used in the network meta-analysis (DOCX file, 13.6 KB). File 4: coding of the network meta-analysis using R (DOCX file, 13.6 KB). [file 5849995.f1.zip › 5849995.f1/File 3. Dataset used in the network meta-analysis..docx]

Supplementary 3. Dataset used in the network meta-analysis.

|  | **Study/Treatment** | **Total Patients** | **Event** | **AI used** |
| --- | --- | --- | --- | --- |
|  | **Heart Failure** |  |  |  |
| **1** | Adriaan Voors, 2017 | 4254 | 1738 | ML |
| **2** | Ashir Javeed, 2020 | 303 | 15 | ML |
| **3** | Bobak Mortazavi, 2016 | 1004 | 299 | ML |
| **4** | Davide Chicco, 2020 | 520 | 299 | ML |
| **5** | Eric Adler, 2020 | 5822 | 1516 | ML |
| **6** | Garrett Bowen, 2018 | 52514 | 10676 | ML |
| **7** | Giulia Lorenzoni, 2019 | 380 | 210 | ML |
| **8** | Rui Chen, 2019 | 62 | 22 | ML |
| **9** | Joon-myoung Kwon, 2019 (1) | 25776 | 25025 | DL |
| **10** | Joon-myoung Kwon, 2019 (2) | 2165 | 2165 | DL |
| **11** | Joon-myoung Kwon, 2019 (3) | 22,765 | 55163 | DL |
| **12** | Oluwarotimi Samuel, 2017 | 297 | 59 | ML |
| **13** | Stephen Weng, 2017 | 24970 | 378,256 | ML |
|  | **Diabetes** |  |  |  |
| **14** | Nam-Kyoo Lim, 2019 | 4566 | 1873 | ML |
| **15** | Saqib Awan, 2019 | 10757 | 2458 | ML |
| **16** | Sarah Cohen, 2021 | 29991 | 78 | ML |
| **17** | Shishir Rao, 2021 | 100,071 | 20606 | DL |
|  | Bobak Mortazavi, 2016 | 1004 | 450 | ML |
|  | Davide Chicco, 2020 | 520 | 125 | ML |
|  | Garrett Bowen, 2018 | 52514 | 35732 | ML |
|  | Rui Chen, 2019 | 62 | 15 | ML |
|  | **Hypertension** |  |  |  |
|  | Nam-Kyoo Lim, 2019 | 5625 | 3589 | ML |
|  | Rui Chen, 2019 | 62 | 25 | ML |
|  | Saqib Awan, 2019 | 10757 | 5497 | ML |
|  | Shishir Rao, 2021 | 60043 | 39427 | DL |
|  | **Stroke** |  |  |  |
|  | Bobak Mortazavi, 2016 | 1004 | 96 | ML |
|  | Saqib Awan, 2019 | 10757 | 1014 | ML |
