# Supplementary material for: Effectiveness of Artificial Intelligence Models for Cardiovascular Disease Prediction: Network Meta-Analysis
Source: Comput Intell Neurosci. 2022 Feb 24;2022:5849995. doi: 10.1155/2022/5849995 (PMC8894073; doi:10.1155/2022/5849995)
Supplement: Supplementary Materials — File 1: QUADAS-2 tool (DOCX file, 13.9 KB). File 2: characteristics of the selected studies (DOCX file, 24.0 KB). File 3: dataset used in the network meta-analysis (DOCX file, 13.6 KB). File 4: coding of the network meta-analysis using R (DOCX file, 13.6 KB). [file 5849995.f1.zip › 5849995.f1/File 4. Coding of the network meta-analysis using R..docx]

**Supplementary 4. Coding of the network meta-analysis using R.**

**mydata=read.csv(file.choose(),header=T)**

**str(mydata)**

**attach(mydata)**

**p2=pairwise(treatment,Event,Total.Patients,studlab = paste(Study),data=mydata,sm="OR")**

**p2**

**mynet=netmeta(p2)**

**mynet**

**forest(mynet, layout = "subgroup", reference.group = "Heart Failure")**

**mymeta=metaprop(Total.Patients,Event, studlab = "Study", data=mydata, method.ci = "WS", method = "Inverse")**

**forest(mymeta, xlab = "symmetric", st)**

**netmeta(mydata)**

**net1=pairwise(list(treat1,treat2,treat3,treat4),list(event1,event2,event3,event4),time=list(year1,year2,year3,year4),studlab = Study,data=mydata,sm="IRR")**

**net1**

**network=netmeta(net1)**

**network**

**summary(net1)**

**netgraph(network,points = TRUE, cex.points = 4,cex = 1.25)**

**tnames <- c("Heartfailure(GBM)","Stroke(SVM)","Diabetes(ANN)","Hypertension(RF)")**

**trts<- c("Heartfailure(GBM)","Stroke(SVM)","Diabetes(ANN)","Hypertension(RF)")**

**netgraph(network, highlight = "Heartfailure:Diabetes",seq=trts)**

**netgraph(network, points = TRUE, cex.points = 4,cex = 1.25, labels = tnames)**

**######not clear####**

**net2 <- netmeta(TE, Std, treat1, treat2,treat3,treat4,data = mydata, sm = "MD", reference = "Heartfailure")**

**netgraph(net2, highlight = "rosi:plac")**

**#########end######**

**forest.netmeta(network, overal.test=TRUE, reference.group=network$Heartfailure(GBM))**

**funnel.netmeta(network)**

**ord <- c("Diabetes", "Heartfailure", "Hypertension", "Stroke")**

**funnel(network, order = ord, col = c("blue","red","green"))**

**netleague(network,seq=netrank(network), ci=FALSE)**

**b=netleague(network,comb.fixed = FALSE,seq=trts,digits = 2)**

**b**

**np <- netposet(network,network,ooutcomes = outcomes)**

**hasse(np)**

**plot(np)**

**write.table(b$random, file = "b-random.csv",row.names = FALSE, col.names = FALSE,sep = ",")**

**#################redo###################**

**####define order of treatment**

**trts <- c("Diabetes", "Heartfailure", "Hypertension", "Stroke")**

**###outcome label**

**outcomes <- c("Prediction with ML", "Prediction with DL")**

**###Prediction with ML**

**p1 <- pairwise(treat = list(treat1, treat2, treat3, treat4),event = list(event1, event2, event3, event4),n = list(n1, n2, n3, n4),studlab = Study, data = mydata, sm = "OR")**

**net1 <- netmeta(p1, comb.fixed = FALSE,seq = trts, ref = "Heartfailure", small = "bad")**

**forest.netmeta(p1)**

**metareg(~ trts,meta1)**

**metareg(~ trts, data=meta1)**

**metareg(meta1)**
